# Antagonism of Therapeutic Agents and What It Teaches

**Published:** 1878-10-20

**Authors:** 


The Antagonism of Therapeutic Agents, and What it Teaches. The essay to which was awarded the Fothergillian Gold Medal of the Medical Society of London for 1878, by J. William Fothergill M D. Edinburg, Member Royal College of Physicians of London etc. *Henry C. Lea, Phil.* A neat little work of 157 pages containg many practical hints for the practioner.

